# Resveratrol prevents inflammation and oxidative stress response in LPS-induced human gingival fibroblasts by targeting the PI3K/AKT and Wnt/β-catenin signaling pathways

**DOI:** 10.1590/1678-4685-GMB-2020-0349

**Published:** 2021-07-02

**Authors:** Lihua Li, Junxiong Li, Yujiao Wang, Xin Liu, Siyu Li, Yan Wu, Wanrong Tang, Ya Qiu

**Affiliations:** 1 North Sichuan Medical College, Department of Dentistry, Nanchong, Sichuan, P.R. China.; 2 University of Chinese Academy of Sciences, Chongqing Savaid Stomatology Hospital, Department of General Dentistry, Chongqing, P.R. China.; 3 Affiliated Hospital of North Sichuan Medical College, Nanchong, Sichuan, P.R. China.

**Keywords:** Resveratrol, PI3K/AKT pathway, Wnt/β-catenin pathway, chronic periodontitis

## Abstract

This study aimed to elucidate the anti-inflammatory and antioxidant properties of resveratrol (RSV) in human gingival fibroblasts (HGFs) following stimulation by *P. gingivalis* lipopolysaccharide (LPS). The levels of the inflammatory cytokines IL-1β, IL-6, IL-8 and TNFα, the activity of the antioxidant enzymes SOD and GSH-Px, and the levels of MDA, were evaluated by ELISA. It was observed that the expression of IL-1β, IL-6, IL-8 and TNFα in LPS-induced HGFs was significantly downregulated by RSV in a dose-dependent manner. RSV also partly increased oxidative stress (OS)-related factors, including SOD and GSH-Px, which was accompanied by a decrease in MDA production, although the results were not statistically significant. Additionally, RSV-induced deactivation of the PI3K/AKT and Wnt/β-catenin pathways in LPS-induced HGFs was observed by western blot analysis. Subsequently, it was demonstrated treatment with PI3K/AKT pathway inhibitor (LY294002) or Wnt/β-catenin pathway inhibitor (Dickkopf-1, DKK-1) could further enhance the anti-inflammatory and antioxidant effects of RSV by downregulating the expression of IL-1β, IL-6, IL-8 and TNFα, and the production of MDA, and increasing the activity of SOD and GSH-Px in LPS-induced HGFs. These results suggested RSV attenuated the inflammation and OS injury of *P. gingivalis* LPS-treated HGFs by deactivating the PI3K/AKT and Wnt/β-catenin signaling pathways.

## Introduction

Chronic periodontitis (CP), a destructive oral disease, is currently the main reason for tooth loss in adults (Slots, 2017). CP is usually characterized by chronic inflammation associated with pathogenic bacteria, such as *Porphyromonas gingivalis* (*P. gingivalis*) in subgingival plaques, resulting in soft tissue destruction, alveolar bone resorption and, eventually, tooth loss (Rovai *et al*., 2016). *P. gingivalis* can stimulate host immune response via lipopolysaccharide (LPS) and the subsequent production of inflammatory cytokines (Zenobia and Hajishengallis, 2015). In particular, the proinflammatory cytokines IL-1β, IL-6, IL-8 and TNFα have been demonstrated to be the most important factors involved in periodontal tissue destruction (Noh *et al*., 2013; Zheng *et al*., 2017).

Human gingival fibroblasts (HGFs), which are mainly present in the gingival connective tissue, play an important role in the formation, regeneration, function implementation and repair of periodontal tissues (Bartold *et al*., 2000). HGFs not only possess an active self-renewal ability, but also synthesize and degrade extracellular matrix components, such as collagen, elastic fibers and glycoproteins (Hoang *et al*., 1997). Of note, HGFs are regulated by immune factors secreted by immune cells. At the same time, HGFs also produce cytokines that finally participate in local inflammation (Naruishi and Nakata, 2018; Shang *et al*., 2018). Furthermore, oxidative stress (OS) occurs when excessive reactive oxygen species (ROS) accumulate and exceed the compensatory antioxidant capacity of the organism (Hernández-Ríos *et al*., 2017). OS leads to destruction of the periodontium, in a direct way (by damaging the biomolecules) or indirect ways [by affecting the production of pro- and antioxidant factors and enzymes, such as superoxide dismutase (SOD), glutathione peroxidase (GSH-Px) and malondialdehyde (MDA)]. Thus, the suppression of inflammation and OS play a key role in the treatment of CP.

Resveratrol (RSV; 3,4',5-trihydroxystilbene) is a natural polyphenol plant antitoxin, which has a variety of biological activities, including antioxidant, anticancer and anti-inflammatory properties, among others (Yousef *et al*., 2017; Yang *et al*., 2018; Sánchez-Melgar *et al*., 2019). It has been reported that RSV improved experimental periodontitis in diabetic mice and inhibited LPS-induced inflammation in gingival epithelial cells (GECs) by negatively regulating TLR4 signaling (Zhen *et al*., 2015). However, the function and mechanism of RSV on LPS-stimulated HGFs *in vitro* remain unclear. More importantly, accumulating evidence suggests that the PI3K/AKT (Lei and Su, 2019) and Wnt/β-catenin signaling pathways (Liu *et al.,* 2018b) are critical mediators in the regulation of cell oxidative stress and are related to the development of CP and other chronic inflammatory diseases. Thus, the present study was undertaken to evaluate the anti-inflammatory and antioxidant effects of RSV in LPS-stimulated HGFs and elucidate the role of the PI3K/AKT and Wnt/β-catenin signaling pathways in the effect of RSV.

## Material and Methods

### Tissue collection and cell culture

Healthy gingival tissues were collected from three systemically healthy donors (12-16 years old) following the extraction of the canine due to orthodontics. Informed written consent was obtained from each subject and their legal guardian. The study protocol was approved by the Ethics Committee of the North Sichuan Medical College. The isolated gingival tissues were stored in Dulbecco’s modified Eagle’s medium (DMEM, PM150220, Procell) with 5% penicillin/streptomycin (Merck KGaA, St. Louis, MO, USA) in an ice bath. Then, the tissues were incubated with DMEM containing 0.05% collagenase I (Sigma-Aldrich, St. Louis, MO, USA) and were digested with 4 mg/mL dispase II (Sigma-Aldrich, St. Louis, MO, USA) for 30 min at 37 °C. After termination of the digestion, the single-cell suspension was seeded into 25 cm^2^ air-permeable flasks and cultured in DMEM supplemented with 20% fetal bovine serum (FBS, cat. no. E600001-0100, Sangon Biotech, Shanghai, China) and 1% penicillin/streptomycin in a humidified incubator with 5% CO_2_ and 37 °C. The culture medium was changed once every 3 days and cells were passaged with 0.25% Trypsin-EDTA (Beijing Solarbio Science & Technology Co., Ltd., Beijing, China) solution until the formation of an 80-90% confluent cell monolayer. The passaged HGFs were then cultured in 10% FBS DMEM without antibiotics. HGFs were used from passage 3 to 5.

### Cell treatment

HGFs plated in 6-well plates were incubated to approximately 80% confluence. *P. gingivalis* LPS (1 𝜇g/mL) was added into the DMEM without FBS for 24 h. Untreated cells were used as a control. For screening optimum concentration, HGFs were treated with RSV (Sigma-Aldrich, St. Louis, MO, USA) for 24 h. In addition, RSV co-transfected HGFs were treated with PI3K/AKT pathway inhibitor (LY294002) or Wnt/β-catenin pathway inhibitor (Dickkopf-1, DKK-1) for 24 h, followed by LPS stimulation for 30 min. The experiment was divided into 5 groups as follows: Control, LPS, LPS+RSV, LPS+RSV+LY294002 and LPS+RSV+DKK-1 groups. The selection of RSV concentration gradient was based on previous studies (Kang *et al*., 2012; Zhang *et al*., 2018).

### Cell viability assay

The viability of HGFs was monitored using a Cell Counting Kit-8 (CCK-8, BS350B, Biosharp) according to the manufacturer’s instructions. Briefly, HGFs were plated at a density of 5x10^3^ cells/well in a 96-well plate and treated with 20, 40 and 80 𝜇mol/L RSV for 24 h. The culture medium of each well was immediately replaced with 100 μL of 10% CCK-8 reagent-DMEM and the cells were incubated for another 2.5 h at 37 °C in CO_2_ incubator. The cell viability was measured using an enzyme linked immune monitor (Thermo Fisher Scientific, Inc., USA) at 450 nm. The experiments were repeated three times. 

### Western blot analysis

HGFs were plated in a 6-well plate at a density of 1x10^6^ cells/well. Then, the cells were treated with RSV or/and LY294002 or DKK-1 for 24 h, followed by LPS stimulation for 30 min. Whole-cell lysates were washed three times in phosphate-buffered saline (PBS) and lysed in RIPA buffer (P0013C, Beyotime Institute of Biotechnology, Shanghai, China) supplemented with 1% phenylmethylsulfonyl fluoride (Beyotime Institute of Biotechnology, Shanghai, China) to obtain the proteins. A bicinchoninic acid (BCA) assay kit (CWBIO, Beijing, China) was used to determine the protein concentration. An equal amount of protein (40 μg) was loaded in each lane and electrophoresed together with molecular weight standards (Bio-Rad Laboratories, Inc., Hercules, CA, USA) in separate lanes on a 10% SDS polyacrylamide gel electrophoresis. Subsequently, the proteins were transferred onto polyvinylidene fluoride (PVDF) membranes (Amersham, Piscataway, NJ, USA) at 300 mA for 110 min. The membranes were blocked with 10% non-fat dry milk for 1 h and incubated with corresponding protein antibodies or a rabbit anti-β-actin monoclonal antibody. Subsequently, the membranes were incubated with a HRP goat anti-rabbit IgG (ab6721, Abcam). The net optical density was analyzed with a gel image processing system (Image-pro Plus 6.0, Media Cybernetics, Sliver Spring, MD). The primary antibodies used were as follows: PI3K (1/1000, cat. no. ab32089, Abcam), AKT (1/10000, cat. no. ab179463, Abcam), p-PI3K (1/500, cat. no. ab182651, Abcam), p-AKT (1/5000, cat. no. ab81283, Abcam), Wnt5a (1/100, cat. no. ab229200, Abcam), β-catenin (1/1000, cat. no. ab16051, Abcam) and β-actin (1/1000, cat. no. AC026, Abclonal). Subsequently, the protein bands were visualized using the ECL system (KF001, Affinity Biosciences) and β-actin was used as a loading control. The experiments were repeated five times.

### Enzyme-linked immunosorbent assay (ELISA)

The contents of IL-1β, IL-6, IL-8, TNFα, SOD, MDA and GSH-Px in culture supernatants were measured with ELISA kits (BioLegend, San Diego, CA, USA) according to the manufacturer’s instructions. Briefly, HGFs were seeded in a 24-well plate at a density of 2x10^5^/well. The cells were treated with RSV and/or LY294002 or DKK-1 for 24 h, followed by subsequent stimulation for LPS for 30 min. Then, the plate was washed 5 times with PBS, and 50 μL biotinylated antibody working solution was added to each well and kept at 37 °C for 20 min. After washing as aforementioned, 100 μL enzyme conjugate working solution was added into each well and kept at 37 °C for 10 min. Then, 100 μL TMB solution was added into each well and kept at 37 °C for 15 min in the dark prior to the addition of 100 μL stopping solution into each well. The optical absorbance values at 450 and 570 nm wavelength were measured using an enzyme linked immune monitor (Thermo Fisher Scientific, Inc., USA). The experiments were repeated five times.

### Statistical analysis

The data are presented as means ± standard deviation (SD). Statistical analysis was performed with the SPSS software (version 20.0, IBM Corp., Chicago, IL, USA). Statistical significance was determined using analysis of variance (one-ANOVA). Differences were considered significant at *P*<0.05. All error bars shown represent the calculated SD across triplicate experiments.

### Ethics approval

The study protocol was approved by the Ethics Committee of the North Sichuan Medical College.

## Results

### Effect of RSV on the proliferation of HGFs induced by LPS

**Figure 1 -:**
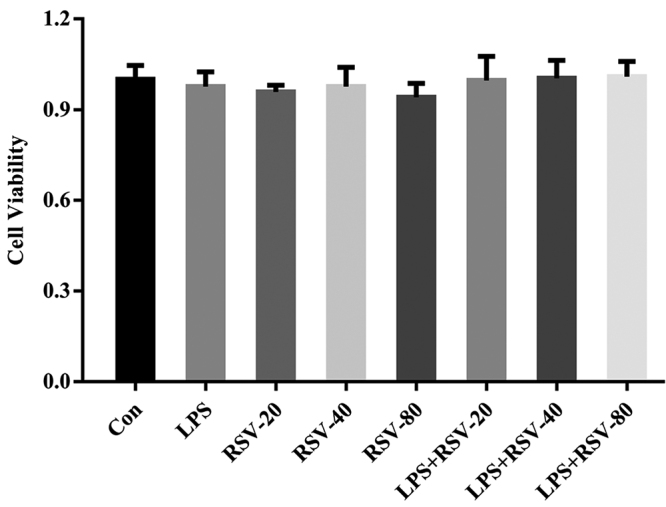
The effect of RSV on the proliferation of HGFs induced by LPS. Cells were cultured with RSV at different final concentration of 20 𝜇mol/L (RSV-20), 40 𝜇mol/L (RSV-40), and 80 𝜇mol/L (RSV-80), respectively, with and without LPS (1 𝜇g/mL) for 24 h. Cell viability was measured by Cell Counting Kit-8 assay. Error bars represent the SD. Statistical significance was determined with one-way ANOVA. RSV, resveratrol; HGF, human gingival fibroblast; LPS, lipopolysaccharide.

Cell viability was detected using the CCK-8 assay. Data revealed that exposure of HGFs to RSV at 20, 40 and 80 𝜇mol/L was not associated with significant cytotoxic effects (Figure 1). These results demonstrated that the effects of RSV on LPS-induced HGFs in the present study are not attributed to non-specific cytotoxicity.

### RSV inhibits inflammation and OS in LPS-stimulated HGFs

**Figure 2 -:**
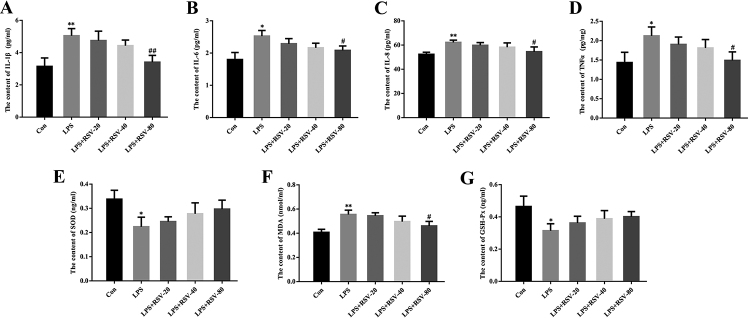
RSV inhibited inflammation and OS in LPS-stimulated HGFs. HGFs were pretreated with 20 (RSV-20), 40 (RSV-40), and 80 μmol/L (RSV-80) RSV for 24 h, followed by LPS (1 𝜇g/mL) stimulation for 24 h. Culture supernatants were collected to evaluate the protein levels of IL-1β (A), IL-6 (B), IL-8 (C), TNFα (D), SOD (E), MDA (F) and GSH-Px (G) by ELISA. Error bars represent SD. Statistical significance was determined with one-way ANOVA. ^*^ *P*<0.05 (vs Control), ^**^ *P*<0.01 (vs Control), ^#^ *P*<0.05 (vs LPS), ^##^ *P*<0.01 (vs LPS). RSV, resveratrol; HGF, human gingival fibroblast; LPS, lipopolysaccharide; OS, oxidative stress; SOD, superoxide dismutase; MDA, malondialdehyde; GSH-Px, glutathione peroxidase.

Next, the anti-inflammatory effects of RSV on LPS-stimulated HGFs were investigated. As presented in Figure 2 A-D, LPS stimulation led to increased levels of IL-1β, IL-6, IL-8 and TNFα, whereas RSV dose-dependently suppressed the production of IL-1β, IL-6, IL-8 and TNFα in LPS-induced HGFs (Figure 2 A-D). The effect of 80 𝜇mol/L RSV treatment was statistically significant (Figure 2 A-D). In addition, ELISA results revealed that LPS stimulation significantly upregulated the content of MDA but downregulated the content of SOD and GSH-Px, which was partly reversed by RSV in a dose-dependent manner (Figure 2 E-G).

### RSV suppresses LPS-induced activation of the PI3K/AKT and Wnt/β-catenin signaling pathways

**Figure 3 -:**
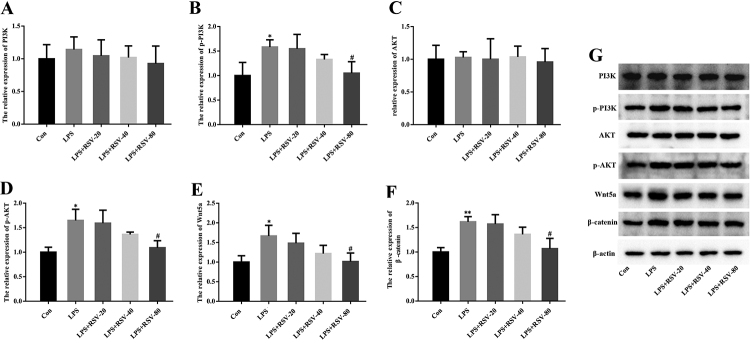
RSV suppressed LPS-induced activation of the PI3K/AKT and Wnt/β-catenin signaling pathways. HGFs were pretreated with 20 (RSV-20), 40 (RSV-40), and 80 μmol/L (RSV-80) RSV for 24 h, followed by LPS (1 𝜇g/mL) stimulation for 24 h. The expression of PI3K, p-PI3K, AKT, p-AKT, Wnt5a and β-catenin was determined by western blot analysis. β-actin served as the loading control. Error bars represent SD. Statistical significance was determined with one-way ANOVA. ^*^ *P*<0.05 (vs Control), ^**^ *P*<0.01 (vs Control), ^#^ *P*<0.05 (vs LPS). RSV, resveratrol; HGF, human gingival fibroblast; LPS, lipopolysaccharide.

To elucidate the mechanism underlying the inhibitory effects of RSV, we investigated the activation of the PI3K/AKT and Wnt/β-catenin signaling pathways, which play important roles in LPS-induced inflammation and OS-related cytokine production. Western blot analysis indicated that LPS significantly promoted the expression of phosphorylated (p-)PI3K, p-AKT, Wnt5a and β-catenin in HGFs (Figure 3B, D-G). As expected, RSV pretreatment (80 𝜇mol/L) markedly decreased the phosphorylation levels of PI3K and AKT, as well as Wnt5a and β-catenin expression levels, in the presence of LPS (Figure 3B, D-G). However, the expression of PI3K and AKT was not changed in all treatment groups (Figure 3A, C, G).

### PI3K/AKT and Wnt/β-catenin pathways mediate the regulatory effects of RSV on inflammation and OS in LPS-stimulated HGFs

**Figure 4 -:**
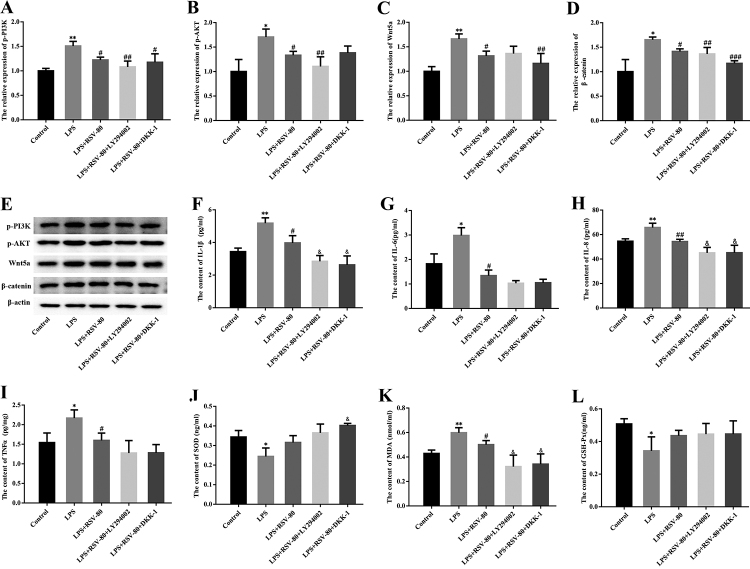
PI3K/AKT and Wnt/β-catenin pathways mediated the regulatory effects of RSV on inflammation and OS of LPS-stimulated HGFs. HGFs were treated with PI3K/AKT pathway inhibitor (LY294002) or Wnt/β-catenin pathway inhibitor (DKK-1), followed by treatment with LPS and/or RSV. (A-E) The expression of p-PI3K, p-AKT, Wnt5a and β-catenin was determined by western blot analysis. β-actin served as the loading control. Culture supernatants were collected to evaluate the protein levels of IL-1β (F), IL-6 (G), IL-8 (H), TNFα (I), SOD (J), MDA (K) and GSH-Px (L) by ELISA. Error bars represent SD. Statistical significance was determined with one-way ANOVA. ^*^ *P*<0.05 (vs Control), ^**^ *P*<0.01 (vs Control), ^#^ *P*<0.05 (vs LPS), ^##^ *P*<0.01 (vs LPS), ^###^ *P*<0.001 (vs LPS), ^&^ *P*<0.05 (vs LPS+RSV-40). RSV, resveratrol; HGF, human gingival fibroblast; LPS, lipopolysaccharide; OS, oxidative stress; SOD, superoxide dismutase; MDA, malondialdehyde; GSH-Px, glutathione peroxidase.

To assess whether the PI3K/AKT and Wnt/β-catenin pathways mediated pro-inflammatory effects and OS cytokine generation in HGFs, HGFs were treated with PI3K/AKT pathway inhibitor (LY294002) or Wnt/β-catenin pathway inhibitor (DKK-1), and the cells were subsequently treated with LPS and/or RSV. Western blot analysis revealed that LY294002 decreased the phosphorylation levels of PI3K and AKT in LPS- and RSV-treated HGFs (Figure 4A, B, E). In addition, DKK-1 decreased Wnt5a and β-catenin expression levels in LPS and RSV-treated HGFs (Figure 4 C-E). The ELISA results revealed that 80 𝜇mol/L RSV induced downregulation of IL-1β, IL-6, IL-8 and TNFα levels, which were partly increased by LY294002 or DKK-1 (Figure 4 F-I). Furthermore, SOD and GSH-Px were upregulated and MDA was downregulated when HGFs were treated with LPS and RSV (Figure 4 J-L). Inhibition of the PI3K/AKT or Wnt/β-catenin pathways in the presence of LPS and RSV further increased the levels of these cytokines (Figure 4 J-L). These findings suggested that RSV inhibited inflammation and OS in LPS-stimulated HGFs partly through inactivation of the PI3K/AKT and Wnt/β-catenin signaling pathways.

## Discussion

Clinical periodontal treatments aimed at removing the adherent plaque bacteria by scaling, curettage and root planing are occasionally supplemented with antibiotics to control the formation of oral microorganisms. Related literature has shown that the active ingredients of natural medicines have beneficial effects on the treatment of CP due to their low toxicity, high efficiency, and the inhibition of bacterial resistance and infection (Li X *et al.,* 2018). In the present study, the therapeutic effects of RSV on LPS-stimulated HGF inflammation were determined. The expression of the proinflammatory cytokines IL-1β, IL-6, IL-8 and TNFα were examined. LPS-stimulated HGFs exhibited higher pro-inflammatory cytokine levels, and RSV treatment significantly reduced the secretion of pro-inflammatory cytokines. To further verify the beneficial effect of RSV on OS injury of LPS-stimulated HGFs, the expression of antioxidant factors, including SOD, GSH-Px and MDA, was measured. It was observed that the LPS-induced decrease in SOD and GSH-Px and increase in MDA levels were partly alleviated by RSV treatment. Moreover, it was revealed that these effects of RSV were exerted through inactivation of the PI3K/AKT and Wnt/β-catenin signaling pathways.

RSV is a non-flavonoid polyphenol compound that contains a stilbene structure and is a natural non-steroidal antioxidant. RSV can be extracted from grape skin, berries and peanuts (Bhat and Pezzuto*,* 2002; Pervaiz, 2003; Pangeni *et al.,* 2014). RSV has a variety of biological activities and pharmacological effects, and its anti-inflammatory and antioxidant effects have been widely studied. A previous study demonstrated that RSV inhibited LPS-induced endothelial adhesion molecule expression and vascular inflammation by inhibiting NF-κB activation in leukocytes (Park *et al.,* 2009). The combination of RSV and silymarin was reported to significantly enhance the viability of fibroblasts, and RSV was able to inhibit LPS-induced IL-6 and IL-8 expression (Shahidi *et al.,* 2017). RSV can attenuate the growth and virulence factor expression of *Staphylococcus aureus*, *Staphylococcus epidermidis*, *Mycobacterium smegmatis* and *P. gingivalis* (Sakagami *et al.,* 2007; Kugaji *et al.,* 2019). Thus, RSV has been considered for use in the treatment of CP. A number of signal transduction pathways and cytokines have been found to be involved in the effects of RSV on *P. gingivalis*-induced inflammation in HGFs and ligature-induced periodontitis animal models. RSV led to alveolar bone resorption and decreased IL-17 expression in rats with experimental periodontitis (Casati *et al.,* 2013). Similar data demonstrated that RSV effectively promoted osteogenesis mainly via SIRT1/FOXO3A signaling and UNX2 gene expression (Tseng *et al.,* 2011). The present study proved that treatment with RSV did not significantly affect HGF viability, and a previous study reported that RSV inhibited the secretion of IL-1β, IL-6, IL-8 and TNFα induced by LPS in HGFs (Buranasin and Mizutani, 2018). 

It is known that OS is considered to be a key factor implicated in cell injury. Excessive oxygen free radical accumulation usually causes lipid peroxidation and protein oxidative damage in the cell membrane, thereby destroying cell membrane integrity and inducing cell inflammation or apoptosis. Several studies suggested that OS mediates the proliferation, apoptosis and migration of HGFs, as well as gingival wound healing (Cheng *et al.,* 2015; Kido *et al.,* 2017; Buranasin and Mizutani, 2018). It was demonstrated that, when HGFs are exposed to LPS/streptococcal cell walls (SCW)/formyl-methionyl-leucyl-phenylalanine, the manganese superoxide dismutase (MnSOD) expression is upregulated (Skaleric *et al.,* 2000). It is known that oxidant and antioxidant factors, such as SOD, GSH-Px and MDA, are crucial OS-related factors, and OS has been implicated in the development and progression of periodontitis (Tonguç *et al.,* 2011; Özdem *et al.,* 2017). The present study revealed elevated MDA level as well as reduced SOD and GSH-Px levels in the model group, whereas RSV partially reversed these changes in expression, indicating that RSV may alleviate oxidative stress in LPS-induced HGFs.

Mechanically, RSV could inhibit cancer cell proliferation and invasiveness via inhibition of the PI3K/AKT and Wnt/β-catenin pathways (Tsai *et al.,* 2013; Liu *et al.,* 2014). Previous reports have revealed that the PI3K/AKT and Wnt/β-catenin pathways are critical mediators during the treatment of periodontal disease (Nakayama *et al.,* 2015; Miranda *et al.,* 2017). In gingival epithelial cells, *P. gingivalis* was shown to regulate PI3K and AKT and the phosphorylation levels of the AKT downstream proteins GSK3, mTOR and Bad, which is linked to cell survival and immune responses (Nakayama *et al*., 2015). It was recently found that LPS from *P. gingivalis* induced autophagy of HGFs via the PI3K/AKT signaling pathway (Liu *et al.,* 2018a). In addition, plantamajoside, caffeic acid phenethyl ester and farrerol all suppressed the inflammatory response in LPS-stimulated HGFs through inhibiting the PI3K/AKT signaling pathway (Wang *et al*., 2016; Li L *et al*., 2017; Liu *et al*., 2019). In an experimental periodontal disease model, gliclazide treatment reduced myeloperoxidase activity, MDA, IL-1β and TNF-α levels via downregulation of PI3K and AKT (Araújo and Morais, 2019). 

Emerging evidence demonstrated that DKK1, an antagonist of Wnt/β-catenin signaling, suppressed bone formation and contributed to inflammation in periodontitis (Miranda *et al.,* 2017). In addition, higher levels of Wnt3a, β-catenin and matrix metalloproteinase inducer (EMMPRIN) were observed in gingival tissues of CP. Furthermore, LPS significantly enhanced β-catenin induction in a human immortalized oral epithelial cell (HIOEC)/HGF co-culture model (Liu *et al*., 2018 b). In addition, in periodontitis, aberrant activation of the Wnt/β-catenin signaling pathway could inhibit the EMMPRIN/MMP-2/ 9 axis (Liu *et al*., 2018b). The Wnt antagonist sFRP1 significantly reduced bone loss of periapical lesions *in vivo* (Yang *et al*., 2021). In human periodontal ligament fibroblasts (hPLFs), knockdown of β-catenin or treatment with DKK1 facilitated H_2_O_2_-induced oxidative damage (Kook *et al*., 2016). Therefore, it was hypothesized that RSV suppressed the inflammation and OS injury in LPS-induced HGFs through attenuating the PI3K/AKT and Wnt/β-catenin signaling pathways. The data of the present study support this hypothesis, as activated PI3K/AKT and Wnt/β-catenin signaling was observed after LPS stimulation. Furthermore, RSV partly inhibited the activation of the PI3K/AKT and Wnt/β-catenin signaling pathways. In HGFs, co-treatment with LPS and RSV, and specific inhibition of PI3K/AKT (LY294002) and Wnt/β-catenin (DKK-1), further reduced inflammation and OS.

## Conclusions

The present study provided evidence and discussed the mechanisms of action of RSV in LPS-stimulated HGFs. RSV was shown to attenuate the inflammatory and OS response in LPS-stimulated HGFs by inhibiting the PI3K/AKT and Wnt/β-catenin signaling pathways. 
